# *trans*-(4-Acetyl­naphth­yl)chlorido­bis(triphenyl­phosphine-κ*P*)nickel(II) dichloro­methane solvate

**DOI:** 10.1107/S1600536808027621

**Published:** 2008-09-06

**Authors:** Yu-Hua Liu, Chen Chen, Lian-Ming Yang

**Affiliations:** a Beijing National Laboratory for Molecular Sciences (BNLMS), Laboratory of New Materials, Institute of Chemistry, Graduate School of the Chinese Academy of Sciences, Beijing 100049, People’s Republic of China; b Beijing National Laboratory for Molecular Sciences (BNLMS), Laboratory of New Materials, Institute of Chemistry, Chinese Academy of Sciences, Beijing 100080, People’s Republic of China

## Abstract

The title compound, [Ni(C_12_H_9_O)Cl(C_18_H_15_P)_2_]·CH_2_Cl_2_, was synthesized from the reaction between 1-acetyl-4-chloro­naphthalene, NiCl_2_·6H_2_O and triphenyl­phosphine (PPh_3_) in ethanol. The compound contains one crystallographically unique nickel ion in a pseudo-square-planar geometry.

## Related literature

For related literature, see: Brandsma *et al.* (1998 ▶); Semmelhack *et al.* (1971 ▶); Soolinger *et al.* (1990 ▶); Chen & Yang (2007 ▶); Cramer & Coulson (1975 ▶); Morrell & Kochi (1975 ▶); Parshall (1974 ▶); Semmelhack & Ryono (1975 ▶); Tsou & Kochi (1979*a* ▶,*b* ▶).
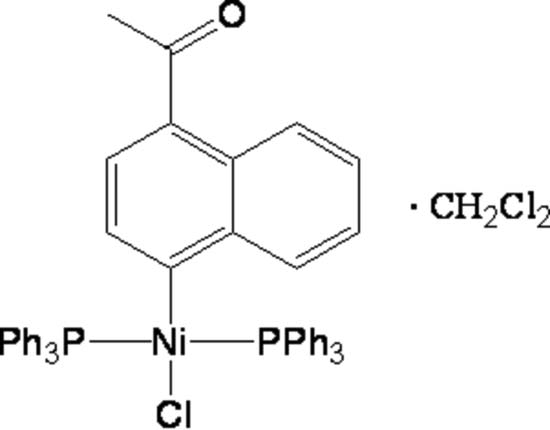


## Experimental

### 

#### Crystal data

[Ni(C_12_H_9_O)Cl(C_18_H_15_P)_2_]·CH_2_Cl_2_
*M* *_r_* = 872.82
Monoclinic, 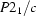

*a* = 21.203 (4) Å
*b* = 10.957 (2) Å
*c* = 21.048 (4) Å
β = 117.95 (3)°
*V* = 4319.5 (15) Å^3^
*Z* = 4
Mo *K*α radiation
μ = 0.74 mm^−1^
*T* = 296 (2) K
0.22 × 0.17 × 0.14 mm

#### Data collection

Rigaku R-AXIS RAPID IP area-detector diffractometer
Absorption correction: multi-scan (*ABSCOR*; Higashi, 1995 ▶ *T*_min_ = 0.853, *T*_max_ = 0.903
14070 measured reflections
7579 independent reflections
4611 reflections with *I* > 2σ(*I*)
*R*_int_ = 0.063

#### Refinement

*R*[*F* ^2^ > 2σ(*F* ^2^)] = 0.080
*wR*(*F* ^2^) = 0.182
*S* = 1.05
7579 reflections
505 parameters
9 restraints
H-atom parameters constrained
Δρ_max_ = 0.39 e Å^−3^
Δρ_min_ = −0.53 e Å^−3^

### 

Data collection: *RAPID-AUTO* (Rigaku, 2001 ▶); cell refinement: *RAPID-AUTO*; data reduction: *RAPID-AUTO*; program(s) used to solve structure: *SHELXS97* (Sheldrick, 2008 ▶); program(s) used to refine structure: *SHELXL97* (Sheldrick, 2008 ▶); molecular graphics: *SHELXTL* (Sheldrick, 2008 ▶); software used to prepare material for publication: *SHELX97*.

## Supplementary Material

Crystal structure: contains datablocks global, I. DOI: 10.1107/S1600536808027621/bv2106sup1.cif

Structure factors: contains datablocks I. DOI: 10.1107/S1600536808027621/bv2106Isup2.hkl

Additional supplementary materials:  crystallographic information; 3D view; checkCIF report

## Figures and Tables

**Fig. 1.**
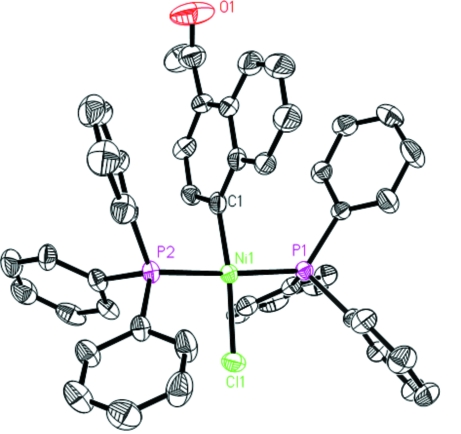
A view of the complex, Ellipsoids are drawn at the 30% probability level.

## supplementary crystallographic information

### Comment

Research in the field of organonickel catalysts has developed significantly in recent years. Semmelhack *et al.* (1971) have demonstrated that the Ni(II)–(σ–aryl) complex may act as an intermediate (oxidative adduct) in the catalytic cycle of Ni-catalyzed cross-couplings. Cramer & Coulson (1975), Morrell & Kochi (1975), Parshall (1974), Tsou & Kochi (1979a) and Tsou & Kochi (1979b)  have also conducted an intensive investigation of Ni(II)–(σ–aryl) complexes, focusing mainly on insight into the nature and mechanism of nickel-catalyzed processes. In addition, Soolinger *et al.* (1990) have shown that it is possible to use such complexes as catalyst in cross-coupling reactions. Consequently, we were interested in the synthesis and direct application of Ni(II)–(σ–aryl) complex catalysts for carbon-heteroatom coupling. In particular, we are investigating a type of isolatable *trans*-haloarylbis(triphenylphosphine)nickel(II) that is readily available and air- and thermally stable (Chen & Yang, 2007). For this purpose, we have  synthesized the title compound in an analogous fashion to a previous literature precedent (Brandsma *et al.* 1998).

The reaction between NiCl_2_^.^6H_2_O, PPh_3_ and 1-acetyl-4-chloronaphthalene in ethanol leads to the formation the title compound (I) in high yield. The Ni^2+^ metal centre of the complex displays a pseudo-square-planar geometry (Figure I).

### Experimental

A stirred mixture of 48.0 g (0.20 mol) of NiCl_2_^.^6H_2_O, 115.3 g (0.44 mol) of triphenylphosphine and 900 ml of 96% ethanol was heated until a gentle reflux started. 1-Acetyl-4-chloronaphthalene (0.4 mol, 82 g, excesss) was then added, followed by zinc dust (13 g, 0.2 mol, Merck, analytical grade) over 5 min. The mixture very soon turned yellow. After stirring and heating under reflux for 1.5 h (under nitrogen), the mixture was cooled to 293 K. Four 20-ml portions of 30% aqueous hydrochloric acid were added over 15 min. After stirring for 1.5 h, the solid was filtered off on a sintered-glass funnel and successively washed with 200 ml of ethanol, twice with 200 ml of 1*M* aqueous hydrochloric acid, twice with 200 ml of 96% ethanol and once with 200 ml of pentane. The yellowish solid was dried *in vacuo*. The yield was at least 80%. Single crystals suitable for X-ray measurements were grown by slow evaporation of a CH_2_Cl_2_ solution and the crystals contain one molecule of CH_2_Cl_2_. 1HNMR (CDCl_3_, 300 MHz): 2.40 (s, 3 H), 5.29 (s, 2 H), 7.11–7.15 (m, 15 H), 7.22–7.25 (m, 8 H), 7.47–7.49 (m, 13 H). Anal. Calcd for C_4_8H_39_ClNiOP_2_?CH_2_Cl_2_: C, 67.43; H, 4.73. Found: C, 67.76; H, 4.71.

### Refinement

All nine restraints were used to make the refinement of the slightly disordered solvent, dichloromethane, more stable. Six of the restraints were used to make the anisotropic displacement parameters of C49 in dichloromethane approximately isotropic. The other three restraints were used to make the components of the anisotropic displacement parameters in the direction of the C-Cl bond in dichloromethane approximately equal. H atoms were fixed geometrically and allowed to ride on their parent atoms, with C—H distances of 0.93–0.97 Å, and with *U*_iso_=1.2–1.5*U*_eq_ of the parent atoms.

### Crystal data

**Table:**

[Ni(C_12_H_9_O)Cl(C_18_H_15_P)_2_]·CH_2_Cl_2_	*F*(000) = 1808
*M**_r_* = 872.82	*D*_x_ = 1.342 Mg m^−^^3^
Monoclinic, *P*2_1_/*c*	Mo *K*α radiation, λ = 0.71073 Å
*a* = 21.203 (4) Å	Cell parameters from 14070 reflections
*b* = 10.957 (2) Å	θ = 2.2–25.0°
*c* = 21.048 (4) Å	µ = 0.75 mm^−^^1^
β = 117.95 (3)°	*T* = 296 K
*V* = 4319.5 (15) Å^3^	Block, yellow
*Z* = 4	0.22 × 0.17 × 0.14 mm

### Data collection

**Table:**

Rigaku R-AXIS RAPID IP area-detector diffractometer	7579 independent reflections
Radiation source: rotating anode	4611 reflections with *I* > 2σ(*I*)
graphite	*R*_int_ = 0.063
ω scans at fixed χ = 45°	θ_max_ = 25.0°, θ_min_ = 2.2°
Absorption correction: multi-scan (ABSCOR; Higashi, 1995	*h* = −25→25
*T*_min_ = 0.853, *T*_max_ = 0.903	*k* = −13→13
14070 measured reflections	*l* = −24→25

### Refinement

**Table:**

Refinement on *F*^2^	Primary atom site location: structure-invariant direct methods
Least-squares matrix: full	Secondary atom site location: difference Fourier map
*R*[*F*^2^ > 2σ(*F*^2^)] = 0.080	Hydrogen site location: inferred from neighbouring sites
*wR*(*F*^2^) = 0.182	H-atom parameters constrained
*S* = 1.05	*w* = 1/[σ^2^(*F*_o_^2^) + (0.074*P*)^2^ + 3.5819*P*] where *P* = (*F*_o_^2^ + 2*F*_c_^2^)/3
7579 reflections	(Δ/σ)_max_ < 0.001
505 parameters	Δρ_max_ = 0.39 e Å^−^^3^
9 restraints	Δρ_min_ = −0.53 e Å^−^^3^

### Special details

**Table:**

Geometry. All e.s.d.'s (except the e.s.d. in the dihedral angle between two l.s. planes) are estimated using the full covariance matrix. The cell e.s.d.'s are taken into account individually in the estimation of e.s.d.'s in distances, angles and torsion angles; correlations between e.s.d.'s in cell parameters are only used when they are defined by crystal symmetry. An approximate (isotropic) treatment of cell e.s.d.'s is used for estimating e.s.d.'s involving l.s. planes.
Refinement. Refinement of *F*^2^ against ALL reflections. The weighted *R*-factor *wR* and goodness of fit *S* are based on *F*^2^, conventional *R*-factors *R* are based on *F*, with *F* set to zero for negative *F*^2^. The threshold expression of *F*^2^ > σ(*F*^2^) is used only for calculating *R*-factors(gt) *etc*. and is not relevant to the choice of reflections for refinement. *R*-factors based on *F*^2^ are statistically about twice as large as those based on *F*, and *R*- factors based on ALL data will be even larger.

### Fractional atomic coordinates and isotropic or equivalent isotropic displacement parameters (Å^2^)

**Table:**

	*x*	*y*	*z*	*U*_iso_*/*U*_eq_	
Ni1	0.30210 (4)	0.08593 (7)	0.15828 (4)	0.0395 (2)	
P1	0.32506 (8)	0.00633 (14)	0.26432 (7)	0.0388 (4)	
P2	0.27230 (8)	0.16467 (14)	0.05070 (8)	0.0425 (4)	
Cl1	0.40230 (8)	0.00498 (17)	0.16800 (9)	0.0618 (5)	
C1	0.2331 (3)	0.1868 (6)	0.1637 (3)	0.0488 (15)	
C2	0.2542 (3)	0.3039 (6)	0.1945 (3)	0.0487 (15)	
H2A	0.3005	0.3299	0.2083	0.058*	
C3	0.2087 (4)	0.3806 (6)	0.2049 (3)	0.0614 (18)	
H3A	0.2253	0.4560	0.2265	0.074*	
C4	0.1373 (3)	0.3475 (7)	0.1834 (4)	0.0587 (18)	
C5	0.0452 (4)	0.1886 (7)	0.1291 (4)	0.073 (2)	
H5A	0.0131	0.2377	0.1358	0.088*	
C6	0.0226 (4)	0.0753 (9)	0.0977 (5)	0.096 (3)	
H6A	−0.0240	0.0498	0.0830	0.115*	
C7	0.0703 (4)	0.0002 (8)	0.0885 (5)	0.087 (3)	
H7A	0.0559	−0.0768	0.0681	0.104*	
C8	0.1382 (3)	0.0388 (6)	0.1093 (3)	0.0507 (16)	
H8A	0.1691	−0.0121	0.1017	0.061*	
C9	0.1628 (3)	0.1510 (6)	0.1413 (3)	0.0490 (15)	
C10	0.1131 (3)	0.2317 (6)	0.1507 (3)	0.0554 (17)	
C11	0.0928 (5)	0.4364 (8)	0.1947 (5)	0.085 (2)	
C12	0.1249 (5)	0.5371 (9)	0.2479 (5)	0.112 (3)	
H12A	0.0876	0.5869	0.2478	0.169*	
H12B	0.1548	0.5861	0.2352	0.169*	
H12C	0.1529	0.5032	0.2950	0.169*	
C13	0.3593 (3)	−0.1497 (5)	0.2738 (3)	0.0409 (13)	
C14	0.3231 (4)	−0.2292 (6)	0.2175 (3)	0.0612 (18)	
H14A	0.2821	−0.2035	0.1771	0.073*	
C15	0.3475 (4)	−0.3463 (7)	0.2209 (4)	0.077 (2)	
H15A	0.3223	−0.3997	0.1830	0.092*	
C16	0.4082 (4)	−0.3856 (7)	0.2789 (5)	0.073 (2)	
H16A	0.4244	−0.4650	0.2807	0.088*	
C17	0.4447 (4)	−0.3064 (7)	0.3344 (4)	0.070 (2)	
H17A	0.4864	−0.3320	0.3740	0.084*	
C18	0.4201 (3)	−0.1883 (6)	0.3324 (3)	0.0511 (15)	
H18A	0.4450	−0.1356	0.3707	0.061*	
C19	0.2542 (3)	−0.0083 (5)	0.2901 (3)	0.0410 (14)	
C20	0.2276 (3)	0.0955 (6)	0.3072 (3)	0.0534 (16)	
H20A	0.2495	0.1703	0.3100	0.064*	
C21	0.1692 (4)	0.0906 (8)	0.3204 (3)	0.065 (2)	
H21A	0.1514	0.1616	0.3303	0.078*	
C22	0.1384 (4)	−0.0189 (9)	0.3187 (4)	0.078 (2)	
H22A	0.0994	−0.0231	0.3275	0.094*	
C23	0.1648 (4)	−0.1226 (8)	0.3042 (5)	0.087 (3)	
H23A	0.1444	−0.1976	0.3045	0.104*	
C24	0.2212 (4)	−0.1174 (6)	0.2891 (4)	0.0649 (19)	
H24A	0.2375	−0.1889	0.2780	0.078*	
C25	0.3928 (3)	0.0981 (5)	0.3373 (3)	0.0432 (14)	
C26	0.4041 (3)	0.0847 (7)	0.4081 (3)	0.0594 (17)	
H26A	0.3787	0.0260	0.4186	0.071*	
C27	0.4523 (4)	0.1571 (8)	0.4619 (4)	0.074 (2)	
H27A	0.4599	0.1469	0.5089	0.089*	
C28	0.4892 (4)	0.2442 (8)	0.4466 (4)	0.079 (3)	
H28A	0.5212	0.2943	0.4832	0.094*	
C29	0.4795 (3)	0.2588 (6)	0.3778 (4)	0.069 (2)	
H29A	0.5050	0.3182	0.3680	0.083*	
C30	0.4314 (3)	0.1846 (6)	0.3229 (4)	0.0549 (17)	
H30A	0.4253	0.1935	0.2764	0.066*	
C31	0.1786 (3)	0.1968 (6)	−0.0129 (3)	0.0531 (16)	
C32	0.1423 (4)	0.2884 (7)	0.0015 (4)	0.0653 (19)	
H32A	0.1655	0.3321	0.0442	0.078*	
C33	0.0719 (4)	0.3167 (9)	−0.0466 (5)	0.088 (3)	
H33A	0.0481	0.3794	−0.0369	0.105*	
C34	0.0382 (4)	0.2491 (10)	−0.1094 (5)	0.095 (3)	
H34A	−0.0090	0.2667	−0.1421	0.114*	
C35	0.0724 (5)	0.1580 (9)	−0.1239 (5)	0.094 (3)	
H35A	0.0490	0.1135	−0.1663	0.113*	
C36	0.1427 (4)	0.1311 (7)	−0.0751 (4)	0.066 (2)	
H36A	0.1658	0.0674	−0.0849	0.080*	
C37	0.2992 (3)	0.0733 (6)	−0.0052 (3)	0.0484 (15)	
C38	0.2945 (4)	−0.0516 (6)	−0.0044 (4)	0.072 (2)	
H38A	0.2819	−0.0885	0.0279	0.086*	
C39	0.3079 (5)	−0.1232 (7)	−0.0505 (4)	0.084 (2)	
H39A	0.3038	−0.2076	−0.0497	0.101*	
C40	0.3272 (4)	−0.0701 (9)	−0.0972 (4)	0.085 (2)	
H40A	0.3355	−0.1180	−0.1290	0.102*	
C41	0.3345 (4)	0.0533 (8)	−0.0972 (4)	0.081 (2)	
H41A	0.3493	0.0893	−0.1280	0.097*	
C42	0.3200 (4)	0.1257 (6)	−0.0517 (4)	0.0623 (19)	
H42A	0.3243	0.2101	−0.0526	0.075*	
C43	0.3157 (3)	0.3125 (5)	0.0609 (3)	0.0474 (15)	
C44	0.2852 (4)	0.4064 (6)	0.0119 (4)	0.0594 (17)	
H44A	0.2415	0.3939	−0.0287	0.071*	
C45	0.3187 (5)	0.5187 (7)	0.0223 (5)	0.081 (2)	
H45A	0.2970	0.5809	−0.0110	0.097*	
C46	0.3827 (5)	0.5386 (8)	0.0806 (5)	0.083 (2)	
H46A	0.4048	0.6144	0.0875	0.100*	
C47	0.4149 (4)	0.4462 (8)	0.1293 (4)	0.072 (2)	
H47A	0.4593	0.4590	0.1689	0.087*	
C48	0.3810 (3)	0.3325 (6)	0.1198 (3)	0.0553 (17)	
H48A	0.4028	0.2706	0.1533	0.066*	
O1	0.0262 (4)	0.4345 (7)	0.1605 (5)	0.145 (3)	
C49	0.1173 (9)	0.8578 (12)	0.4912 (7)	0.222 (8)	
H49A	0.0664	0.8529	0.4592	0.266*	
H49B	0.1347	0.9313	0.4789	0.266*	
Cl2	0.1563 (2)	0.7385 (4)	0.4758 (3)	0.1911 (17)	
Cl3	0.1305 (3)	0.8714 (6)	0.5768 (3)	0.247 (3)	

### Atomic displacement parameters (Å^2^)

**Table:**

	*U*^11^	*U*^22^	*U*^33^	*U*^12^	*U*^13^	*U*^23^
Ni1	0.0434 (4)	0.0430 (4)	0.0364 (4)	0.0042 (3)	0.0222 (3)	0.0058 (3)
P1	0.0431 (8)	0.0412 (9)	0.0335 (8)	0.0034 (7)	0.0192 (6)	0.0030 (7)
P2	0.0488 (9)	0.0426 (9)	0.0389 (8)	−0.0022 (7)	0.0228 (7)	0.0058 (7)
Cl1	0.0528 (9)	0.0820 (13)	0.0607 (10)	0.0167 (9)	0.0350 (8)	0.0139 (9)
C1	0.047 (3)	0.050 (4)	0.044 (3)	−0.003 (3)	0.018 (3)	0.014 (3)
C2	0.044 (3)	0.050 (4)	0.052 (4)	−0.008 (3)	0.022 (3)	0.001 (3)
C3	0.065 (4)	0.057 (5)	0.055 (4)	0.014 (3)	0.022 (3)	0.002 (3)
C4	0.048 (4)	0.065 (5)	0.063 (4)	0.014 (3)	0.026 (3)	0.016 (4)
C5	0.056 (4)	0.065 (5)	0.087 (6)	0.000 (4)	0.025 (4)	0.008 (4)
C6	0.053 (5)	0.098 (7)	0.126 (8)	−0.005 (5)	0.033 (5)	0.011 (6)
C7	0.062 (5)	0.081 (6)	0.101 (6)	−0.006 (5)	0.023 (4)	0.010 (5)
C8	0.045 (3)	0.048 (4)	0.054 (4)	−0.002 (3)	0.019 (3)	0.002 (3)
C9	0.054 (4)	0.047 (4)	0.046 (3)	−0.001 (3)	0.023 (3)	0.009 (3)
C10	0.055 (4)	0.054 (4)	0.060 (4)	0.014 (3)	0.029 (3)	0.017 (3)
C11	0.076 (6)	0.080 (6)	0.098 (6)	0.010 (5)	0.041 (5)	0.001 (5)
C12	0.127 (8)	0.099 (7)	0.110 (7)	0.037 (6)	0.054 (6)	−0.019 (6)
C13	0.048 (3)	0.037 (3)	0.043 (3)	0.007 (3)	0.026 (3)	0.003 (3)
C14	0.065 (4)	0.060 (5)	0.053 (4)	0.011 (4)	0.023 (3)	−0.001 (3)
C15	0.100 (6)	0.065 (5)	0.072 (5)	0.011 (5)	0.046 (5)	−0.012 (4)
C16	0.091 (6)	0.049 (5)	0.097 (6)	0.020 (4)	0.058 (5)	0.005 (4)
C17	0.064 (4)	0.064 (5)	0.081 (5)	0.018 (4)	0.034 (4)	0.020 (4)
C18	0.052 (4)	0.048 (4)	0.055 (4)	0.007 (3)	0.027 (3)	0.007 (3)
C19	0.046 (3)	0.048 (4)	0.033 (3)	0.011 (3)	0.022 (3)	0.002 (3)
C20	0.064 (4)	0.058 (4)	0.041 (3)	0.010 (3)	0.026 (3)	0.005 (3)
C21	0.072 (4)	0.086 (6)	0.048 (4)	0.023 (4)	0.038 (3)	0.003 (4)
C22	0.072 (5)	0.101 (7)	0.085 (6)	−0.005 (5)	0.055 (5)	−0.002 (5)
C23	0.100 (6)	0.072 (6)	0.121 (7)	−0.030 (5)	0.079 (6)	−0.012 (5)
C24	0.081 (5)	0.055 (5)	0.081 (5)	−0.010 (4)	0.056 (4)	−0.006 (4)
C25	0.043 (3)	0.041 (3)	0.038 (3)	0.011 (3)	0.014 (3)	−0.002 (3)
C26	0.060 (4)	0.070 (5)	0.045 (4)	0.009 (4)	0.022 (3)	−0.003 (4)
C27	0.067 (5)	0.095 (6)	0.043 (4)	0.006 (5)	0.012 (4)	−0.020 (4)
C28	0.051 (4)	0.080 (6)	0.074 (6)	0.012 (4)	0.003 (4)	−0.037 (5)
C29	0.051 (4)	0.052 (4)	0.089 (6)	0.000 (3)	0.020 (4)	−0.012 (4)
C30	0.050 (4)	0.051 (4)	0.058 (4)	0.005 (3)	0.021 (3)	−0.002 (3)
C31	0.057 (4)	0.054 (4)	0.050 (4)	−0.009 (3)	0.026 (3)	0.014 (3)
C32	0.058 (4)	0.071 (5)	0.061 (4)	0.009 (4)	0.023 (4)	0.017 (4)
C33	0.065 (5)	0.096 (7)	0.098 (7)	0.016 (5)	0.035 (5)	0.032 (6)
C34	0.059 (5)	0.104 (8)	0.087 (7)	−0.009 (5)	0.006 (5)	0.036 (6)
C35	0.071 (6)	0.100 (7)	0.079 (6)	−0.025 (5)	0.008 (5)	−0.001 (5)
C36	0.061 (4)	0.062 (5)	0.061 (5)	−0.014 (4)	0.016 (4)	0.005 (4)
C37	0.059 (4)	0.047 (4)	0.040 (3)	−0.007 (3)	0.024 (3)	0.001 (3)
C38	0.113 (6)	0.055 (5)	0.066 (5)	−0.010 (4)	0.059 (5)	−0.002 (4)
C39	0.131 (7)	0.048 (5)	0.080 (6)	0.003 (5)	0.055 (5)	−0.001 (4)
C40	0.094 (6)	0.089 (7)	0.083 (6)	−0.004 (5)	0.051 (5)	−0.022 (5)
C41	0.095 (6)	0.092 (7)	0.078 (5)	−0.023 (5)	0.058 (5)	−0.015 (5)
C42	0.078 (5)	0.060 (5)	0.065 (4)	−0.012 (4)	0.047 (4)	−0.009 (3)
C43	0.059 (4)	0.043 (4)	0.049 (4)	−0.010 (3)	0.032 (3)	−0.005 (3)
C44	0.077 (4)	0.045 (4)	0.061 (4)	−0.002 (4)	0.037 (4)	0.010 (3)
C45	0.099 (6)	0.055 (5)	0.106 (7)	−0.009 (4)	0.062 (6)	0.014 (4)
C46	0.097 (7)	0.058 (5)	0.121 (8)	−0.017 (5)	0.072 (6)	−0.010 (5)
C47	0.059 (4)	0.088 (6)	0.083 (5)	−0.029 (4)	0.045 (4)	−0.041 (5)
C48	0.053 (4)	0.065 (5)	0.061 (4)	−0.009 (3)	0.038 (3)	−0.004 (3)
O1	0.083 (5)	0.133 (6)	0.204 (8)	0.034 (4)	0.054 (5)	−0.043 (6)
C49	0.32 (2)	0.117 (10)	0.143 (9)	0.080 (11)	0.034 (12)	−0.023 (9)
Cl2	0.154 (3)	0.179 (4)	0.273 (5)	0.019 (3)	0.128 (3)	−0.007 (3)
Cl3	0.246 (5)	0.310 (7)	0.184 (4)	−0.016 (5)	0.100 (4)	−0.079 (4)

### Geometric parameters (Å, °)

**Table:**

Ni1—C1	1.880 (6)	C23—C24	1.375 (10)
Ni1—P2	2.2204 (17)	C23—H23A	0.9300
Ni1—Cl1	2.2215 (17)	C24—H24A	0.9300
Ni1—P1	2.2273 (17)	C25—C30	1.375 (8)
P1—C19	1.829 (6)	C25—C26	1.402 (8)
P1—C13	1.831 (6)	C26—C27	1.367 (9)
P1—C25	1.834 (6)	C26—H26A	0.9300
P2—C43	1.825 (6)	C27—C28	1.366 (11)
P2—C37	1.828 (6)	C27—H27A	0.9300
P2—C31	1.836 (6)	C28—C29	1.374 (10)
C1—C9	1.392 (8)	C28—H28A	0.9300
C1—C2	1.412 (8)	C29—C30	1.390 (9)
C2—C3	1.371 (8)	C29—H29A	0.9300
C2—H2A	0.9300	C30—H30A	0.9300
C3—C4	1.409 (9)	C31—C36	1.370 (9)
C3—H3A	0.9300	C31—C32	1.382 (9)
C4—C10	1.420 (9)	C32—C33	1.389 (9)
C4—C11	1.452 (10)	C32—H32A	0.9300
C5—C10	1.374 (9)	C33—C34	1.386 (12)
C5—C6	1.381 (11)	C33—H33A	0.9300
C5—H5A	0.9300	C34—C35	1.350 (12)
C6—C7	1.386 (12)	C34—H34A	0.9300
C6—H6A	0.9300	C35—C36	1.389 (10)
C7—C8	1.362 (9)	C35—H35A	0.9300
C7—H7A	0.9300	C36—H36A	0.9300
C8—C9	1.381 (8)	C37—C38	1.373 (9)
C8—H8A	0.9300	C37—C42	1.373 (8)
C9—C10	1.459 (8)	C38—C39	1.378 (10)
C11—O1	1.248 (9)	C38—H38A	0.9300
C11—C12	1.490 (11)	C39—C40	1.360 (11)
C12—H12A	0.9600	C39—H39A	0.9300
C12—H12B	0.9600	C40—C41	1.361 (11)
C12—H12C	0.9600	C40—H40A	0.9300
C13—C18	1.368 (8)	C41—C42	1.385 (10)
C13—C14	1.378 (8)	C41—H41A	0.9300
C14—C15	1.373 (9)	C42—H42A	0.9300
C14—H14A	0.9300	C43—C48	1.376 (8)
C15—C16	1.363 (10)	C43—C44	1.384 (8)
C15—H15A	0.9300	C44—C45	1.385 (9)
C16—C17	1.367 (10)	C44—H44A	0.9300
C16—H16A	0.9300	C45—C46	1.355 (11)
C17—C18	1.388 (9)	C45—H45A	0.9300
C17—H17A	0.9300	C46—C47	1.374 (11)
C18—H18A	0.9300	C46—H46A	0.9300
C19—C24	1.380 (8)	C47—C48	1.404 (9)
C19—C20	1.390 (8)	C47—H47A	0.9300
C20—C21	1.391 (9)	C48—H48A	0.9300
C20—H20A	0.9300	C49—Cl2	1.658 (13)
C21—C22	1.359 (10)	C49—Cl3	1.696 (15)
C21—H21A	0.9300	C49—H49A	0.9700
C22—C23	1.362 (11)	C49—H49B	0.9700
C22—H22A	0.9300		
			
C1—Ni1—P2	88.62 (18)	C23—C22—H22A	120.0
C1—Ni1—Cl1	165.33 (19)	C22—C23—C24	120.7 (7)
P2—Ni1—Cl1	92.97 (7)	C22—C23—H23A	119.7
C1—Ni1—P1	88.87 (18)	C24—C23—H23A	119.7
P2—Ni1—P1	176.57 (7)	C23—C24—C19	121.5 (7)
Cl1—Ni1—P1	90.05 (7)	C23—C24—H24A	119.3
C19—P1—C13	103.0 (3)	C19—C24—H24A	119.3
C19—P1—C25	103.2 (3)	C30—C25—C26	118.9 (6)
C13—P1—C25	107.8 (3)	C30—C25—P1	120.4 (5)
C19—P1—Ni1	120.53 (18)	C26—C25—P1	120.6 (5)
C13—P1—Ni1	111.32 (19)	C27—C26—C25	120.6 (7)
C25—P1—Ni1	110.0 (2)	C27—C26—H26A	119.7
C43—P2—C37	105.7 (3)	C25—C26—H26A	119.7
C43—P2—C31	103.2 (3)	C28—C27—C26	119.8 (7)
C37—P2—C31	101.2 (3)	C28—C27—H27A	120.1
C43—P2—Ni1	109.7 (2)	C26—C27—H27A	120.1
C37—P2—Ni1	114.7 (2)	C27—C28—C29	120.8 (7)
C31—P2—Ni1	120.8 (2)	C27—C28—H28A	119.6
C9—C1—C2	118.0 (6)	C29—C28—H28A	119.6
C9—C1—Ni1	123.6 (5)	C28—C29—C30	119.8 (8)
C2—C1—Ni1	118.3 (4)	C28—C29—H29A	120.1
C3—C2—C1	122.3 (6)	C30—C29—H29A	120.1
C3—C2—H2A	118.8	C25—C30—C29	120.0 (7)
C1—C2—H2A	118.8	C25—C30—H30A	120.0
C2—C3—C4	121.4 (6)	C29—C30—H30A	120.0
C2—C3—H3A	119.3	C36—C31—C32	118.3 (6)
C4—C3—H3A	119.3	C36—C31—P2	121.5 (6)
C3—C4—C10	118.4 (6)	C32—C31—P2	120.2 (5)
C3—C4—C11	117.3 (7)	C31—C32—C33	121.4 (8)
C10—C4—C11	124.3 (6)	C31—C32—H32A	119.3
C10—C5—C6	122.8 (8)	C33—C32—H32A	119.3
C10—C5—H5A	118.6	C34—C33—C32	118.3 (9)
C6—C5—H5A	118.6	C34—C33—H33A	120.9
C5—C6—C7	119.3 (8)	C32—C33—H33A	120.9
C5—C6—H6A	120.4	C35—C34—C33	121.2 (8)
C7—C6—H6A	120.4	C35—C34—H34A	119.4
C8—C7—C6	120.1 (8)	C33—C34—H34A	119.4
C8—C7—H7A	120.0	C34—C35—C36	119.6 (8)
C6—C7—H7A	120.0	C34—C35—H35A	120.2
C7—C8—C9	122.3 (7)	C36—C35—H35A	120.2
C7—C8—H8A	118.9	C31—C36—C35	121.2 (8)
C9—C8—H8A	118.9	C31—C36—H36A	119.4
C8—C9—C1	121.0 (6)	C35—C36—H36A	119.4
C8—C9—C10	118.3 (6)	C38—C37—C42	118.4 (6)
C1—C9—C10	120.7 (6)	C38—C37—P2	119.4 (5)
C5—C10—C4	123.5 (7)	C42—C37—P2	122.1 (5)
C5—C10—C9	117.3 (7)	C37—C38—C39	121.2 (7)
C4—C10—C9	119.1 (6)	C37—C38—H38A	119.4
O1—C11—C4	122.7 (8)	C39—C38—H38A	119.4
O1—C11—C12	116.2 (8)	C40—C39—C38	119.8 (7)
C4—C11—C12	121.1 (8)	C40—C39—H39A	120.1
C11—C12—H12A	109.5	C38—C39—H39A	120.1
C11—C12—H12B	109.5	C39—C40—C41	119.8 (8)
H12A—C12—H12B	109.5	C39—C40—H40A	120.1
C11—C12—H12C	109.5	C41—C40—H40A	120.1
H12A—C12—H12C	109.5	C40—C41—C42	120.4 (8)
H12B—C12—H12C	109.5	C40—C41—H41A	119.8
C18—C13—C14	119.3 (6)	C42—C41—H41A	119.8
C18—C13—P1	123.0 (5)	C37—C42—C41	120.2 (7)
C14—C13—P1	117.6 (4)	C37—C42—H42A	119.9
C15—C14—C13	120.1 (6)	C41—C42—H42A	119.9
C15—C14—H14A	120.0	C48—C43—C44	118.3 (6)
C13—C14—H14A	120.0	C48—C43—P2	119.1 (5)
C16—C15—C14	121.1 (7)	C44—C43—P2	122.6 (5)
C16—C15—H15A	119.4	C43—C44—C45	121.1 (7)
C14—C15—H15A	119.4	C43—C44—H44A	119.5
C15—C16—C17	118.9 (7)	C45—C44—H44A	119.5
C15—C16—H16A	120.6	C46—C45—C44	120.6 (8)
C17—C16—H16A	120.6	C46—C45—H45A	119.7
C16—C17—C18	120.8 (7)	C44—C45—H45A	119.7
C16—C17—H17A	119.6	C45—C46—C47	119.5 (8)
C18—C17—H17A	119.6	C45—C46—H46A	120.2
C13—C18—C17	119.8 (6)	C47—C46—H46A	120.2
C13—C18—H18A	120.1	C46—C47—C48	120.4 (7)
C17—C18—H18A	120.1	C46—C47—H47A	119.8
C24—C19—C20	116.6 (6)	C48—C47—H47A	119.8
C24—C19—P1	123.5 (5)	C43—C48—C47	120.1 (7)
C20—C19—P1	119.7 (5)	C43—C48—H48A	120.0
C19—C20—C21	121.8 (7)	C47—C48—H48A	120.0
C19—C20—H20A	119.1	Cl2—C49—Cl3	115.4 (8)
C21—C20—H20A	119.1	Cl2—C49—H49A	108.4
C22—C21—C20	119.4 (7)	Cl3—C49—H49A	108.4
C22—C21—H21A	120.3	Cl2—C49—H49B	108.4
C20—C21—H21A	120.3	Cl3—C49—H49B	108.4
C21—C22—C23	120.0 (7)	H49A—C49—H49B	107.5
C21—C22—H22A	120.0		
			
C1—Ni1—P1—C19	32.3 (3)	Ni1—P1—C19—C24	103.8 (5)
P2—Ni1—P1—C19	−10.5 (13)	C13—P1—C19—C20	163.7 (4)
Cl1—Ni1—P1—C19	−162.3 (2)	C25—P1—C19—C20	51.6 (5)
C1—Ni1—P1—C13	153.0 (3)	Ni1—P1—C19—C20	−71.5 (5)
P2—Ni1—P1—C13	110.2 (12)	C24—C19—C20—C21	−2.0 (9)
Cl1—Ni1—P1—C13	−41.6 (2)	P1—C19—C20—C21	173.7 (4)
C1—Ni1—P1—C25	−87.5 (3)	C19—C20—C21—C22	2.0 (9)
P2—Ni1—P1—C25	−130.4 (12)	C20—C21—C22—C23	−0.1 (11)
Cl1—Ni1—P1—C25	77.8 (2)	C21—C22—C23—C24	−1.9 (13)
C1—Ni1—P2—C43	80.4 (3)	C22—C23—C24—C19	1.9 (13)
Cl1—Ni1—P2—C43	−85.1 (2)	C20—C19—C24—C23	0.1 (10)
P1—Ni1—P2—C43	123.2 (12)	P1—C19—C24—C23	−175.4 (6)
C1—Ni1—P2—C37	−160.9 (3)	C19—P1—C25—C30	−142.6 (5)
Cl1—Ni1—P2—C37	33.7 (2)	C13—P1—C25—C30	108.9 (5)
P1—Ni1—P2—C37	−118.1 (12)	Ni1—P1—C25—C30	−12.7 (5)
C1—Ni1—P2—C31	−39.4 (3)	C19—P1—C25—C26	35.0 (5)
Cl1—Ni1—P2—C31	155.2 (3)	C13—P1—C25—C26	−73.6 (5)
P1—Ni1—P2—C31	3.4 (13)	Ni1—P1—C25—C26	164.8 (4)
P2—Ni1—C1—C9	92.1 (5)	C30—C25—C26—C27	0.6 (9)
Cl1—Ni1—C1—C9	−171.5 (4)	P1—C25—C26—C27	−177.0 (5)
P1—Ni1—C1—C9	−85.6 (5)	C25—C26—C27—C28	0.7 (11)
P2—Ni1—C1—C2	−90.3 (4)	C26—C27—C28—C29	−1.2 (11)
Cl1—Ni1—C1—C2	6.2 (11)	C27—C28—C29—C30	0.3 (11)
P1—Ni1—C1—C2	92.0 (4)	C26—C25—C30—C29	−1.5 (9)
C9—C1—C2—C3	2.0 (9)	P1—C25—C30—C29	176.1 (5)
Ni1—C1—C2—C3	−175.7 (5)	C28—C29—C30—C25	1.0 (10)
C1—C2—C3—C4	−1.7 (10)	C43—P2—C31—C36	125.6 (5)
C2—C3—C4—C10	0.2 (10)	C37—P2—C31—C36	16.4 (6)
C2—C3—C4—C11	−178.2 (7)	Ni1—P2—C31—C36	−111.4 (5)
C10—C5—C6—C7	1.2 (13)	C43—P2—C31—C32	−54.9 (6)
C5—C6—C7—C8	−0.9 (13)	C37—P2—C31—C32	−164.1 (5)
C6—C7—C8—C9	1.5 (12)	Ni1—P2—C31—C32	68.1 (6)
C7—C8—C9—C1	178.3 (6)	C36—C31—C32—C33	−2.1 (10)
C7—C8—C9—C10	−2.1 (9)	P2—C31—C32—C33	178.4 (6)
C2—C1—C9—C8	178.6 (6)	C31—C32—C33—C34	1.1 (11)
Ni1—C1—C9—C8	−3.8 (8)	C32—C33—C34—C35	−0.1 (13)
C2—C1—C9—C10	−1.0 (8)	C33—C34—C35—C36	0.1 (14)
Ni1—C1—C9—C10	176.7 (4)	C32—C31—C36—C35	2.1 (10)
C6—C5—C10—C4	−179.6 (7)	P2—C31—C36—C35	−178.3 (6)
C6—C5—C10—C9	−1.8 (11)	C34—C35—C36—C31	−1.2 (12)
C3—C4—C10—C5	178.5 (6)	C43—P2—C37—C38	159.7 (5)
C11—C4—C10—C5	−3.3 (11)	C31—P2—C37—C38	−93.0 (6)
C3—C4—C10—C9	0.8 (9)	Ni1—P2—C37—C38	38.7 (6)
C11—C4—C10—C9	179.0 (6)	C43—P2—C37—C42	−24.5 (6)
C8—C9—C10—C5	2.2 (9)	C31—P2—C37—C42	82.8 (6)
C1—C9—C10—C5	−178.3 (6)	Ni1—P2—C37—C42	−145.5 (5)
C8—C9—C10—C4	−179.9 (6)	C42—C37—C38—C39	−2.0 (11)
C1—C9—C10—C4	−0.4 (9)	P2—C37—C38—C39	174.0 (6)
C3—C4—C11—O1	158.6 (9)	C37—C38—C39—C40	0.9 (13)
C10—C4—C11—O1	−19.7 (13)	C38—C39—C40—C41	1.2 (13)
C3—C4—C11—C12	−20.7 (11)	C39—C40—C41—C42	−2.2 (13)
C10—C4—C11—C12	161.1 (8)	C38—C37—C42—C41	1.0 (10)
C19—P1—C13—C18	−99.9 (5)	P2—C37—C42—C41	−174.8 (6)
C25—P1—C13—C18	8.8 (6)	C40—C41—C42—C37	1.0 (12)
Ni1—P1—C13—C18	129.5 (5)	C37—P2—C43—C48	−93.3 (5)
C19—P1—C13—C14	82.8 (5)	C31—P2—C43—C48	160.9 (5)
C25—P1—C13—C14	−168.5 (5)	Ni1—P2—C43—C48	30.9 (5)
Ni1—P1—C13—C14	−47.8 (5)	C37—P2—C43—C44	87.1 (6)
C18—C13—C14—C15	0.9 (10)	C31—P2—C43—C44	−18.7 (6)
P1—C13—C14—C15	178.3 (6)	Ni1—P2—C43—C44	−148.7 (5)
C13—C14—C15—C16	−1.1 (12)	C48—C43—C44—C45	−1.4 (10)
C14—C15—C16—C17	0.2 (12)	P2—C43—C44—C45	178.2 (5)
C15—C16—C17—C18	0.8 (12)	C43—C44—C45—C46	0.8 (12)
C14—C13—C18—C17	0.1 (9)	C44—C45—C46—C47	0.6 (12)
P1—C13—C18—C17	−177.1 (5)	C45—C46—C47—C48	−1.3 (12)
C16—C17—C18—C13	−1.0 (11)	C44—C43—C48—C47	0.6 (9)
C13—P1—C19—C24	−20.9 (6)	P2—C43—C48—C47	−179.0 (5)
C25—P1—C19—C24	−133.0 (5)	C46—C47—C48—C43	0.8 (10)
